# To Tame a Songbird: The Genomics of the Domestication Syndrome in a Songbird Model Species

**DOI:** 10.21203/rs.3.rs-4921127/v1

**Published:** 2025-04-11

**Authors:** Madza Farias-Virgens, David Peede, Terrence Deacon, Kazuo Okanoya, Stephanie A. White, Emilia Huerta-Sanchez

**Affiliations:** 1 Interdepartmental Graduate Program in Molecular, Cellular and Integrative Physiology, University of California Los Angeles; 2 Department of Ecology, Evolution, and Organismal and Evolutionary Biology, Brown University; 3 Center for Computational Molecular Biology, Brown University; 4 Institute at Brown for Environment and Society, Brown University; 5 Department of Anthropology, University of California Berkeley; 6 Graduate School of Arts and Sciences, The University of Tokyo & RIKEN-Brain Science Institute; 7 Department of Integrative Biology and Physiology, University of California Los Angeles; † Moved to Department of Biology, University of Washington

## Abstract

Many domesticated animals share a syndromic phenotype marked by a suite of traits that include more variable patterns of coloration, reduced stress, aggression, and altered risk-taking and exploratory behaviors relative to their wild counterparts. Roughly 150 years after Darwin’s pioneering insight into this phenomenon, reasonable progress has been made in understanding the evolutionary and biological basis of the so-called domesticated phenotype in mammals. However, the extent to which these processes are paralleled in non-mammalian domesticates is scant. Here, we address this knowledge gap by investigating the genetic basis of the domesticated phenotype in the Bengalese finch, a songbird frequently found in pet shops and a popular animal model in the study of learned vocal behaviors. Using whole-genome sequencing and population genomic approaches, we identify strain-specific selection signals in the BF and its wild munia ancestor. Our findings suggest that, like in mammals, the evolution of the domestication syndrome in avian species involves a shift in the selective regime, capable of altering brain circuits favoring the dynamic modulation of motivation and reward sensitivity over overall augmented aggression and stress responses.

## Introduction

Heightened stress and aggressive responses are significant traits of adaptive value for wild animals, manifested as impulsive reactions to perceived threats, often associated with high arousal states [1]. Domesticated animals show lower levels of reactive aggression than their wild counterparts and more consistently exhibit risk-taking behaviors in contexts where they are highly motivated, following spontaneous learned associations and/or as reinforced by domestication practices [2]. These changes are possible due to the attenuation of environmental sources of selection commonly found in the wild and often accompanied by enhanced selective pressures imposed by close socialization with individuals of the same or distinct species in the domestic setting [3].

**Fig. 1.**
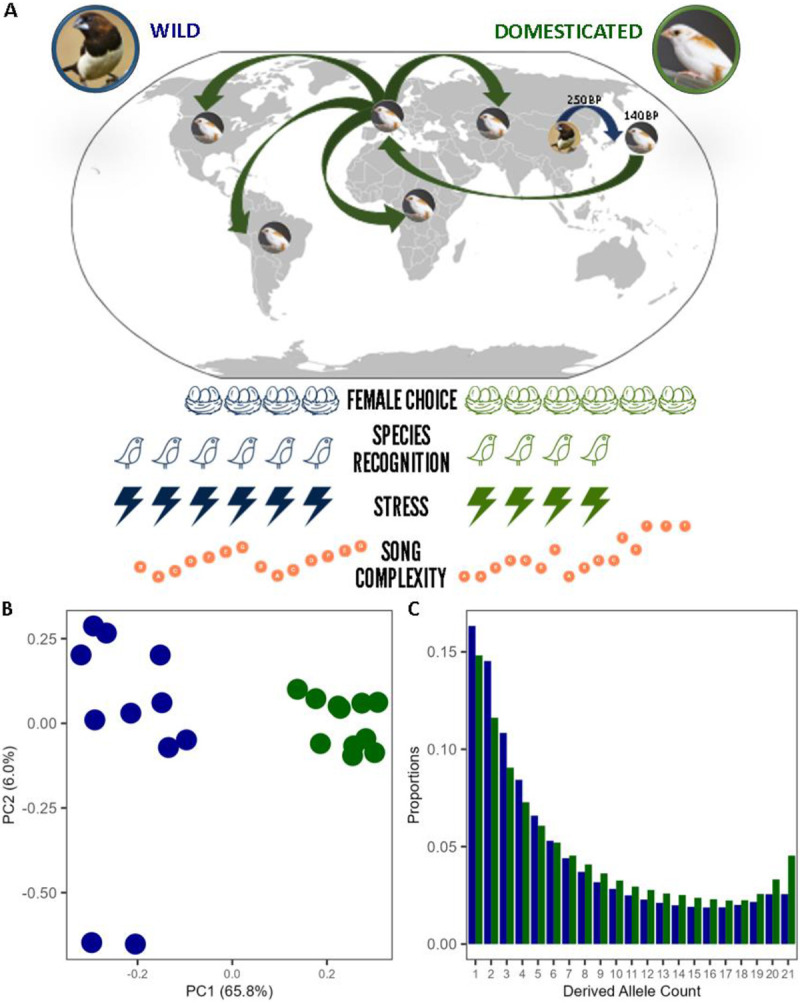
The WBM (left; wild; blue) and the BF (right; domesticated; green).(A) Environmental variables and their relationship with stress and birdsong complexity in the wild and domesticated scenarios. (B) PCA of whole genomic genotype likelihoods in WBM and BF. (C) BF and WBM unfolded Site Frequency Spectrums.

Here, we study one such domestication case in an under-examined order, the passerine lineage. Beginning ~250YBP, white-backed munias (WBM; *Lonchura striata*), an extant wild songbird readily found throughout East Asia, were brought to Japan by aviculturists [4]. Shortly after the first century of domestication, these artificially bred WBMs started to exhibit piebald (i.e., patchy) patterns of plumage coloration, marking the origins of today’s Bengalese finches (BF; *Lonchura striata domestica*) [5] (Fig. 1A). Since then, BFs have become popular cage birds, known for their easy socialization and willingness to foster offspring, including those of other songbird species [6]. In addition to confirming BFs’ attenuated stress response, evidenced by decreased reactive aggression and neophobia relative to their munia ancestors [7-9], studies show that adult BFs’ song retains a greater degree of variability in the ordering of vocal elements than exhibited by WBM [10, 11]. These traits evolved in concert in the BF, characterizing a prototypical case of the domestication syndrome in the avian clade.

While we have amassed considerable knowledge about the behavioral, physiological, and morphological differences between domesticated and wild mammals [12] and posed specific evolutionary hypotheses for how those changes came about [13, 14], the extent to which this is paralleled in the non-mammalian realm is under-studied. In this work, we address this gap in knowledge by investigating the genetic basis of the domesticated phenotype in the BF. Using whole-genome sequencing data and population genomic approaches, we identified strain-specific and overlapping selection signals in the domesticate BF and its wild munia ancestor and interrogated annotated genes within those regions of interest (ROIs). Selection signals in the WBM population are associated with morphological and behavioral traits relating to stress and aggression. These selection signals are weaker or absent in the domesticated BF, which instead shows signals related to reward aspects of social interactions and feeding. In accordance with comparisons drawn between other domesticated species and their wild counterparts [15], our findings suggest that the evolution of the domestication syndrome in the BF likely involved the attenuation of selective forces associated with hormonal regulation of stress and aggression and the intensification of ones leading to dynamic modulation of motivation and reward sensitivity.

## Methods

### Sample collection

Blood samples were collected from 15 BFs and 15 WBMs housed in the Okanoya Lab at RIKEN-Brain Science Institute (RIKEN-BSI, Japan). BF source colonies in the Okanoya lab originated from and are continuously supplied by various breeders in Japan and diligently maintained per standard bird breeder practices to avoid undesired effects of inbreeding. WBMs were imported from multiple wild colonies from different locations in Taiwan and bred in Okanoya Lab’s aviaries, following similar practices as for BFs (~5 generations at the time of sample collection for this study). To avoid immediate relatedness biases, we selected individuals with no less than two degrees related to one another within each BF and WBM population. Blood samples were extracted by puncturing the brachial vein and collecting the resulting droplet in a capillary tube, a well-suited method for sampling small birds with minimal injury and distress. Samples were stored at −80°C at RIKEN-BSI until transported to UC Berkeley under a material transfer agreement and with USDA-APHIS approval (#128913). Housing conditions and procedures conformed to RIKEN's Animal Care and Use Committee.

### Whole genome library preparation and sequencing

Total DNA was extracted from individual blood samples using a DNAeasy kit (Qiagen, Germantown, MD) following protocols at the Evolutionary Genetics Laboratory at UC Berkeley. DNA quality was visually inspected by gel electrophoresis, and DNA concentrations were measured using NanoDrop spectrophotometry and a Qubit dsDNA BR Assay Kit (Thermo Fisher Scientific, Waltham, MA). Samples showing signs of degradation or yielding DNA concentrations insufficient for library preparation (< 50ng/microL) were discarded. Whole genomic double-stranded DNA from the remaining 13 BF and 12 WBM DNA samples were fragmented to a 350bp average size using a Bioruptor sonicator (Diagenode-Hologic, Denville, NJ). Sheared samples were visualized by gel electrophoresis to verify size distribution, and DNA concentrations were re-measured using a Qubit dsDNA BR Assay Kit. All 25 samples passed this quality control step and were used to prepare whole-genome libraries using a KAPA Hyper Prep PCR-free Kit (Hoffmann-La Roche, Basel, Switzerland) and TruSeq adapters (Illumina, San Diego, CA). Library sizing information was obtained by electrophoresis using an Agilent 2100 Bioanalyzer system (Agilent Technologies, Santa Clara, CA). The 11 BF and 11 WBM libraries passing this quality control step were sequenced on 7 lanes in a 4000 HiSeq system (Illumina, San Diego, CA) in two replicates at UC Berkeley’s QB3 sequencing facility.

### Sequencing data preprocessing and quality control

The resulting paired-end sequencing reads (150bp) underwent quality-control procedures using the software package *readcleaner* (github.com/tplinderoth/ngsQC), which includes trimming adapters using *cutadapt* v1.10; pair-end read merging using *pear* v0.9.10; filtering contaminants identified through alignment to the human (GRCh38) and *E. coli* (ASM584v2) genomes using *bowtie2* v2.2.9 [16], and generates final read quality reports using *fastqc* v0.11.4 [17] (Supp. Text 1).

Clean reads were aligned to the recently published, high-coverage zebra finch (ZF; *Taeniopygia guttata*) genome sequenced as part of the Vertebrate Genomes Project (bTaeGut2.pat.W.v2-Pacific Biosciences, Menlo Park, CA) using the *Burrows-Wheeler Alignment* (BWA) tool. Genome indexes for each reference genome were generated using *BWA* v0.7.17 [18] and *SAMtools* v.10 [19], along with sequence dictionaries from *Picard Tools* v2.0.1 (Picard Tools: CreateSequenceDictionary), and aligned using appropriate read group information (Supp. Text 1).

After alignment, duplicate reads were marked (Picard Tools: MarkDuplicates), and an additional step of local indel realignment was performed using *GATK* v3.8.0 [20]. Indels were called from all sampled individuals (GATK: HaplotypeCaller) and used to create lists of indel sites for each sampled individual separately (GATK: RealignerTargetCreator), which were then used as targets in the realignment (GATK: IndelRealigner) (Supp. Text 1).

A subsequent filtering step was performed to remove paralogous or repetitive sequence regions that confound short-read mapping using *ngsParalog* (github.com/tplinderoth/ngsParalog). This step involved calling single nucleotide polymorphisms (SNPs) in each BF and WBM population separately using *Analysis of Next Generation Sequencing Data* (ANGSD) [21], a software package that calculates various population genetic summary statistics from genotype likelihoods (Supp. Text 1). The union of the identified variant positions (angsd:-*dovcf 1*) in each population served in estimating per-site paralog-log likelihood ratios. Likelihood ratios for each site were then compared between BF and WBM, and the highest values were retained. The −log likelihood ratio is asymptotically distributed as a 50:50 mixture of a chi-square with one degree of freedom and a chi-square distribution with zero degrees of freedom. Accordingly, the cutoff above which a given site would be considered paralogous and excluded from future analyses was calculated as 0.5+0.5∗qchisq(p,df=1)=1−W, where W=0.05 significance level, and corrected for multiple hypothesis testing using the total number of sites.

After excluding potential paralogous or repetitive regions, we calculated depth counts in each remainder position for each BF and WBM individual (angsd: *-doCounts 1, -dumpCounts 2*) (Supp. Text 1). The sequencing data rendered an average per-site coverage depth per individual of 8x. To avoid spurious results from missing data, sites covered less than 9 out of 11 individuals in each population were further filtered from the data. Additionally, sites with average depth across individuals of > 20x (~2SD+) in each population were filtered out, as they could be sequencing artifacts or represent paralogous and repetitive regions bypassed by the prior filtering. Finally, among the remaining sites, only diallelic sites were inferred from in downstream analyses.

### Population genomic analyses

Using the *GATK* model within *ANGSD,* we calculated genotype likelihoods and per-site allele frequencies (Supp. Text 1). Genotype likelihoods from sequencing reads were calculated as the probability of the observed sequencing data X given the three different possible genotypes g=0,1,2 representing the number of copies of the minor allele in a site for an individual, P(X∣G=g). The allele frequency at a given site in the population was used in calculating the prior genotype probability of the same site for an individual, $P(G_{is}is|p_{s})$, which was calculated under the assumption of Hardy-Weinberg Equilibrium (HWE) for the three different possible genotypes g=0,1,2 as $P(G_{is}=0|p_{s})={\left(1-p_{s}\right)}^{2}$, $P(G_{is}=1|p_{s})=2p_{s}(1-p_{s})$ and $P(G_{is}=2|p_{s})=P_{s}^{2})$, and used to define posterior genotype probabilities using Bayes’ theorem.

To perform *Principal Component Analysis* (PCA) of the genetic relationship among individuals in our populations, we used a heuristic approach implemented in *PCAngsd* [22] (Supp. Text 1) that does not rely on the assumption of conditional independence between sampled individuals and, in this way, can model missing data with the inferred population structure. *PCAngsd* replaces population allele frequencies with individual allele frequencies calculated from posterior expectations of genotypes to update prior information in calculating posterior genotype probabilities. The individual allele frequencies are computed through a *Singular Value Decomposition* method, where genotypes are reconstructed using the top principal components and serve to inform prior genotype probabilities, assuming HWE in calculating posterior genotype probabilities and updating genotype expectations. This procedure is iterated until individual allele frequencies converge. Finally, the covariance matrix is calculated within a probabilistic framework by summing over individual’s genotype weighted by the joint posterior probability of individual allele frequencies. Eigendecomposition of the updated estimated covariance matrix is then performed to obtain the principal components.

Per site allelic frequencies estimated for each population separately were used to estimate the proportion of alleles at specific frequencies in each population, with ancestral states as defined in the ZF genome – 1-dimensional *Site Frequency Spectrum* (1D-SFS), as well as at a particular frequency in one population and at a different frequency in the other population −2-dimensional SFS (2D-SFS) (Supp. Text 1). Accordingly, considering a set of n aligned DNA sequences, the SFS is defined as the vector that describes the frequency of k=1 to n derived alleles; $\eta ={\left(\eta_{k}\right)}_{k}=0,\ldots ,n$ where $\eta_{k}$ is the number of sites with k derived alleles.

A series of statistics summarizing the variation or distribution of alleles within the dataset was calculated using *ANGSD realSFS.* The 2D-SFS served as the prior in subsequent per site *Fixation index* (Fst) calculations, a commonly used population genetics statistic that measures genetic variance *between* relative to *within* populations. Per-site Fst estimates were calculated using Bathia *et al* [23] derivation of Hudson’s Fst estimator, as implemented in *ANGSD realSFS fst.* In the analysis of multiple SNPs comprising genomic regions, Fst was calculated as the ratio of the sum of per site Bathia et al. estimations of the variance *within* and *between* populations. This approach allowed us to calculate weighted Fst between our BF and WBM populations in non-overlapping windows spanning 10kb across the genome, as well as across all positions within a given gene’s full-length and within exonic and intronic annotated boundaries (Supp. Text 1 and 2; Supp. Data 1).

Unfolded genome-wide 1D-SFSs were used as priors in calculating various estimators describing the genetic diversity in each BF and WBM population separately, using *ANGSD realSFS saf2theta* (Supp. Text 1). These estimators measure the ratio between the nucleotide diversity observed in a population and the expected under a neutral evolution model. We calculated *Waterson’s theta* (tW), *Pairwise theta* (tP), and *Tajima’s D* (TajD) estimators of genetic diversity [24, 25] (Supp. Text 2) for each BF and WBM population in non-overlapping windows spanning 10kb across the genome, as well as across all positions within a given gene’s full-length and within exonic and intronic annotated boundaries (Supp. Data 2).

Selection of a sequence variant associated with an individual’s increased chances of survival and reproduction frequently leads to surrounding variants also increasing in frequency within the population due to linkage disequilibrium (i.e., selective sweep) [26]. An event like this will cause TajD to become negative, as there will be relatively more high-frequency variants than expected under a neutral model, thus rendering the expectation of tP smaller than the expectation of tW. Therefore, one of the general signatures of selection is a loss of nucleotide diversity around a beneficial genetic variant. This effect might result from positive selection during the sweep-up of the newly advantageous variant, or purifying selection, resulting in the purging of deleterious variants surrounding the neutral one (i.e., background selection). Positive values of *Tajima’s D* arise from an excess of intermediate frequency alleles and can result from population bottlenecks, structure, and/or balancing selection.

We performed selection scans using *SweepFinder2* [27], an implementation of a method that performs a composite likelihood ratio test for positive selection (Supp. Text 1). In this method, the likelihood of the null hypothesis is calculated for each population from their respective genome-wide 1D-SFS, and the likelihood of the alternative hypothesis is calculated from a model in which a recent selective sweep alters the neutral spectrum. *SweepFinder2* selection scans were run in non-overlapping windows spanning 10kb each (Supp. Data 3).

## Results

### Differentiation and variation along Bengalese finch and white-backed munia genomes

The PCA of all BF and WBM individuals shows a clear separation between the two populations along PC1 (Fig. 1B). This distinction is marked by a greater proportion of low-frequency genetic variants in the wild WBM population. In contrast, variants at higher-to-fixed frequencies prevail in the domesticated BF, indicating an overall loss of genetic variability within their population compared to their wild counterparts (Fig. 1C).

**Fig. 2.**
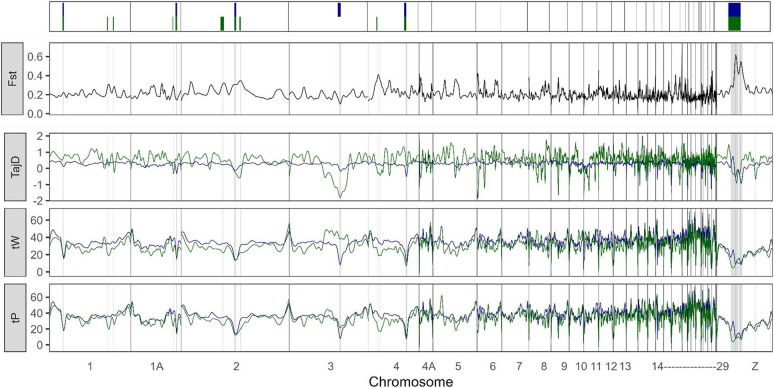
Genetic differentiation and variability in WBM (wild; blue) and BF (domesticated; green) populations. (**top to bottom**) Schematic representation of defined ROIs enclosing sweep signals discussed in this study; 10kb window measures of differentiation between the two populations, calculated as the averaged Fst across all positions within the window; 10kb window estimations of nucleotide diversity, calculated as the Tajima’s D (TajD), Waterson’s theta (tW) and Pairwise theta (tW) across all positions within the window.

Differences in allelic frequencies between BF and WBM are unevenly distributed across windows along individual chromosomes, allowing for the identification of local signals of differentiation between the two songbirds, as measured by the canonical *Fixation index* (Fst) (Fig. 2). While the genetic differentiation of windows within individual autosomes is close to the autosomal average (10kb Windowed Mean Fst +/− SD ~ 0.2±0.08), the sex chromosome Z (ChrZ) shows considerably higher levels of differentiation (10kb Windowed Mean Fst +/− SD ~ 0.3 ±0.15). The higher differentiation of ChrZ may be partly attributed to this chromosome’s smaller effective population size due to its heterogametic mode of transmission (males: ZZ; females: ZW) [28].

We also report genomic windows and sets of genes for which observed variability deviates from expected under neutrality in each population, as evidenced by *Tajima’s D* (TajD), *Waterson’s theta* (tW), and *Pairwise theta* (tP) summary statistics (Fig. 2). Genetic diversity in WBMs deviates less across their genome (WBM autosomal 10kb Windowed Mean TajD +/− SD 0.3±0.43, var = 0.19; WBM ChrZ 10kb Windowed Mean TajD +/− SD 0.2±0.60, var = 0.36), whereas in the BF, long stretches of the genome show either considerable loss or gain of variability (BF autosomal 10kb Windowed Mean TajD +/− SD 0.5±0.78, var = 0.61; BF ChrZ 10kb Windowed Mean TajD +/− SD 0.5±0.82, var = 0.67) (Fig. 2). As with many domesticates, these genome-wide observations are likely due to the heightened effects of genetic drift, set off by the primary population bottleneck that marks the major domestication event [29] and furthered by domestication practices of breeding small BF populations in captivity.

### Regions of interest (ROIs) in the Bengalese finch and the white-backed munia genomes

**Fig. 3.**
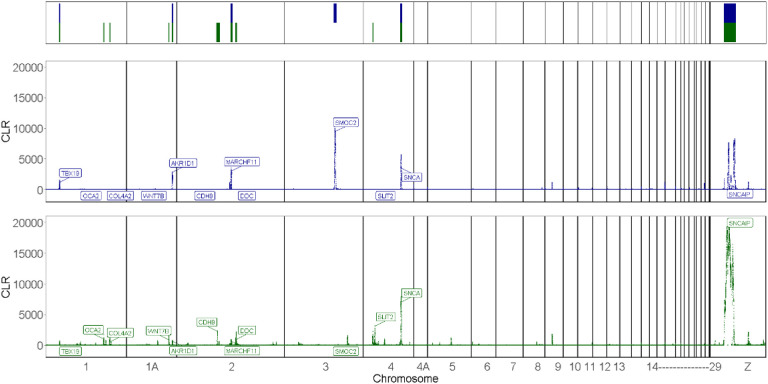
Selection signals in WBM (wild; blue) and BF (domesticated; green) populations. (**top to bottom**) Schematic representation of defined ROIs enclosing sweep signals discussed in this study; selection signals in WBM and BF populations. Each data point represents a window’s composite likelihood of a selective sweep (CLR). Labels highlight the genes within ROIs referred to in the main text.

Our sweep scans allowed the detection of strain-specific signals in the domesticate BF and its wild munia ancestor (Fig. 3). Subsequently, we defined genomic regions of interest (ROIs) as the contiguous segments containing the top 5% of windows with a higher composite likelihood ratio (CLR) in each BF and WBM (Fig. 3; Supp. Table 1). Six ROIs were defined encompassing sweep signals found in four autosomes as exclusive to the BF population: **BF1-Chr1, BF2-Chr1, BF3-Chr1A, BF4-Chr2, BF5-Chr2, BF6-Chr4** (Supp. Fig. 1-12). The genetic differentiation between BF and WBM in these regions was particularly higher than found for the rest of the autosome genome, as shown by their greater-than-autosomal average Fst values (Fst ~ 0.4±0.09, greater than 97% of autosomal windows). TajD values for windows spanning each BF ROI were generally lower for the BF population (10kb Windowed Mean TajD +/− SD ~ −1.0±0.5, lower than 95% of the autosomal windows) than found in the WBM population (10kb Windowed Mean TajD ~ 0.2±0.43; lower than 60% of the autosomal windows), with tW (BF tW ~ 18.7±7.54, lower than 88% of the autosomal windows vs. WBM tW ~33.7±9.44, lower than 54% of the autosomal windows) and tP (BF tP ~ 13.9±5.95, lower than 95% of the autosomal windows vs. WBM tP ~ 35.9±10.40, lower than 56% of the autosomal windows) following the same trend of lower values in the BF, consistent with their greater loss of variability in those genomic segments in the BF. These results suggest that BF ROIs might have experienced substantial changes due to recent or ongoing positive selection specific to the BF population, resulting in higher differentiation with the WBM population.

In the WBM population, we defined four genomic ROIs encompassing sweep signals found in four autosomes (Fig. 3; Supp. Table 1). Among those, only one signal was exclusive to the WBM population, **WBM4-Chr3,** while overlapping signals were also detected in the BF population at **WBM1-Chr1, WBM2-Chr1A,** and **WBM3-Chr2** (Supp. Fig. 1-12). In general, the genetic differentiation of these ROIs is not particularly higher than found in the rest of the autosome genome, as shown by their near-average values of Fst (10kb Windowed Mean Fst +/− SD ~ 0.20±0.09, higher than 53% of the autosomal windows). These aspects suggest that selective sweeps in these regions may have occurred in the ancestral WBM population. Alternatively, these overlapping signals could be false positives, detected by *SweepFinder2* in both populations due to their shared demographic history. Both these scenarios can influence gene function in these regions, with their differing degrees of genetic differentiation resulting from population-specific factors post-divergence, including continued gene flow into and between BF populations, and the different environmental conditions and consequent selective pressures ancestral and derived populations are uniquely subjected to.

TajD values for windows overlapping ROIs **WBM1-Chr1, WBM2-Chr1A,** and **WBM3-Chr2** were lower for the WBM relative to the BF population (WBM TajD ~ −0.4±0.58, lower than 94% of the autosomal windows vs. BF TajD ~ 0.1±0.63, lower than 73% of the autosomal windows), consistent with a greater loss of relative variability in the given genomic region in the WBM population. However, **WBM4-Chr3** ROI presented a different pattern of genetic variation, whereby the WBM population showed an exclusive sweep signal but higher TajD values than the BF population. Closer inspection reveals that TajD values in the BF population decay (BF TajD ~ - 2.0±0.20) and gradually reverse toward less negative values along **WBM4-Chr3,** while TajD values remain closer to 0 in the WBM population (WBM TajD ~ −0.3±0.63) (Supp. Fig. 1-12). The decrease in TajD values in the BF population is accompanied by increases in tW (BF tW ~ 36.5±6.26, greater than 72% of the autosomal windows) and decreases in tP (BF tP ~ 18.9±3.49, lower than 89% of the autosomal windows) suggesting the accumulation of many rare variants rather than common variants in the region in the BF; while in the WBM both tW and tP values remain proportionally lower (WBM tW ~ 5.8±2.22 and tP ~ 5.4±2.56, both lower than 99% of the autosomal windows). This region is associated with a common fragile site prone to mutate and frequently undergoes large structural rearrangements in mammals and birds, within which breakpoints locate to the Parkinson’s disease (PD) associated gene, **PRKN** [30]. Accordingly, our findings of greater variability in the BF population on a region otherwise showing an increased proportion of higher frequency variants in the WBM may relate to increased genomic instability at **PRKN** in the BF population.

As for **WBM4-Chr3** ROI, **BF7-Chr4** ROI presented a unique pattern of genetic variation, whereby the BF population showed a stronger sweep signal but higher TajD values than the WBM population. (BF TajD ~ 0.16±0.72; WBM TajD ~ −0.62±0.65) (Supp. Fig. 1-12). Accordingly, tW and tP values in the BF deviate less from each other along **BF7-Chr4** (BF tW ~ 5.9±3.45, BF tP ~ 6.4±4.28), while WBM shows a more skewed distribution of tW and tP values (WBM tW ~ 10.2±5.29, WBM tP ~ 8.9±5.49). One possible explanation for the genetic pattern we observed is that complex demographic or selective events after a genetic sweep helped shape it. Previous findings support the idea that the BF population experienced a recent bottleneck followed by a rapid expansion [29]. We suggest that the more intense genetic sweep signal in the BF population could result from advantageous alleles quickly rising in frequency during the population growth, while common domestication practices involving the interbreeding of small captive populations, together with changes in selective pressures due to domestication, may have caused selection for genetic diversity to increase at the same or nearby loci experiencing a sweep.

Sweep signals within ChrZ cover a broad genomic area, including a major peak shared by both WBM and BF, **BF8-ChrZ,** flanked by regions showing increased CLR values only in the BF (Supp. Fig. 1-12). **BF8-ChrZ** shows a high degree of differentiation between BFs and WBMs (Fst ~ 0.5±0.17), but similarly negative TajD values (BF TajD −0.3±0.80; WBM TajD −0.1±0.87), suggesting that while both populations have experienced a selective sweep, the specific alleles under selection might differ, leading to distinct genetic patterns in each population.

Our subsequent investigation focused on the genes annotated within these ROIs, enabling us to identify candidates accounting for the reported phenotypic distinctions between BFs and WBMs (Table 1). A number of these genes exhibit selection signals in domesticated avian and mammalian species, spanning various indigenous and enhanced breeds worldwide (Supp. Table 1).

### Selection signals in the WBM population are associated with hormonal regulation of stress and aggression.

Like other domesticates, BFs exhibit less stress than their wild counterparts, as shown by lower levels of cortisol measured in fecal samples collected throughout the day; and lower reactive aggression, as demonstrated by less frequent biting responses when provoked with a stick attached to a piezo-electric sensor [9, 30]. In mammals and birds, stress and aggression are closely linked to hormonal regulation in the body, primarily mediated by cortisol, adrenaline, and testosterone [31]. As a result, genes associated with hormone function are likely to contribute to variations in stress and aggression between domesticated and wild animals [32]. Following this rationale and considering previous genetic associations, we have identified genes implicated in reactive aggression and stress, exhibiting selection signals in wild munias: **TBX19** (**WBM1-Chr1**), **AKR1D1** (**WBM2-Chr1A**), **MARCHF11** (**WBM3-Chr2**). Noticeably, **MARCHF11** shows high divergence in 11 *Lonchura* munia species radiated throughout Australia and Papua New Guinea [33]; specifically, it shows high differentiation in four pairwise comparisons between sympatric *Lonchura* species with minimal genome-wide divergence, therefore possibly involved in generating adaptive diversity in munias in the wild.

We have also identified a selection signal exclusive to the WBM population in a region of importance for craniofacial morphology, for which changes are associated with domestication in mammals [34, 35]. The sweep signal within **WBM4-Chr3** ROI includes the gene **SMOC2,** which codes for a protein related to matrix assembly and cell adhesiveness, expressed in cartilage and bone during development and in the adult skeleton. In mammals, this gene product is implicated in craniofacial dysmorphism, specifically brachycephaly (i.e., shortening of the anteroposterior skull length and widening along the cranial mediolateral axis) and brachygnathism (i.e., underbite) [34-36]. The role of **SMOC2** in craniofacial morphology appears to be preserved in birds, as it figures among genes showing significant SNP association to beak shape and with a high degree of local linkage disequilibrium [37]. Though direct morphological comparisons between BF and WBM are yet to be explored, the absence in the BF of selective signals found in their munia ancestor at **SMOC2** could indicate craniofacial differences between the two. This finding is consistent with controlled observations that WBMs show stronger biting to provocation than BFs, as differences in beak dimensions and/or head width are the major predictors of bite force in finches [38]. Differences in bite force between BF and WBM, and possibly associated craniofacial allometric scaling, could derive from the lack of selective pressures such as physical endurance against aggression and foraging harder and more varied materials and food in the wild.

### Selection signals in the BF population locate to regions of importance to color variation, neurodevelopment, and dopaminergic neuromodulation of behavior.

Eye and coat color tend to be fixed traits within wild species, with some variation related to age and sexual dichromatism [39]. In contrast, individual color variation in the adult stage is characteristic of many domestic species [40]. In line with this, plumage pigmentation is a noticeable difference between BFs and their munia ancestors. While WBMs stereotypically have dark-brown heads, tails, and wing plumage, which morphs into more visibly barred feathers on a darker to lighter gradient towards their flank and rump, BFs’ plumage assumes the most varied combination of tones, with variable body distribution, including dark brown (chocolate), lighter brown (chestnut), yellowish brown tan (fawn), pale yellow (crèmino) and white (albino) morphs. We found selection signals specific to the BF at the **HERC2-OCA2-GABRB3** gene cluster in Chr1 (**BF1-Chr1**). **OCA2** encodes the melanosomal transmembrane protein P, involved in the trafficking and processing of tyrosinase, a catalyzer of crucial steps in the melanogenesis pathway, whose absence halts melanin production [41]. **OCA2** expression is regulated by an element located within an intronic region of its contiguous upstream neighbor **HERC2,** where variants are major determinants of blue/brown iris coloration and are associated with differences in skin pigmentation in humans [42]. Mutations in **OCA2**’s mouse orthologue (i.e., the pink-eyed dilution locus), causing the lack of functional P protein, lead to a pink-eyed and light fur phenotype [41]. Contiguously downstream of **OCA2** is the gene **GABRB3,** which encodes the γ3 subunit of GABAA receptors. Consistent with its major role in neural function and development, mutations in this gene have been frequently associated with the neurodevelopmental aspects of Angelman and Prader-Willi syndromes (AS and PWS, respectively) [43]. However, a potential contribution of **GABRB3** to ocular function and development is suspected, as the deletion of **GABRB3** alone causes a nearly complete loss of retinal pigmentation due to atrophied melanosomes [44]. It has been, therefore, proposed that **GABRB3** or elements within the gene might regulate **OCA2** expression, and impairment of this function may indirectly cause ocular hypopigmentation and visual defects, as seen in PWS in AS. Historical accounts indicate that piebald plumage coloration spontaneously emerged ~140YBP and was picked up by Japanese breeders, who further developed many BF color morphs [4]. Noticeably, BF domestication was marked by significant breeding efforts for full white morphs, leading to the development of the black-eyed and pink-eyed albino BF strains [4]. Our findings raise the possibility of **HERC2-OCA2-GABRG3** gene cluster involvement in BF plumage and eye coloration, which is especially relevant in the context of the development of the pink-eyed albino and other light-colored BF strains.

Several lines of evidence demonstrate BFs have evolved more complex vocal abilities than WBMs: adult BF song retains a greater degree of variability in the ordering of vocal elements than exhibited by WBM; BFs exposed to multiple tutors compose their songs from a combination of excerpts from the different tutor’s songs, in contrast to their wild ancestors, who latch onto and copy the song of only one tutor; moreover, in cross-fostering experiments, BFs learn their WBM foster parent’s song more efficiently than WBMs learn their BF foster parent’s song [10, 11, 45]. Several genes located within the specified BF ROIs exert a significant influence on the regulation of cell guidance and connectivity, particularly relevant in neurodevelopment, and thus potentially contributing to the behavioral signatures observed in the BF: **COL4A2** and **COL4A1** (**BF2-Chr1**), **WNT7B** (**BF3-Chr1A**), **CDH9** (**BF4-Chr2**), **SLIT2** (**BF6-Chr4**). We also uncovered clear signals of selection exclusive to the BF population in regions including genes capable of eliciting changes in behavior through their involvement in neuromodulation, particularly in relation to a direct association with dopaminergic transmission: **DDC (BF5-Chr2).** Additionally, we detected major selection signals in the BF in regions enclosing two other genes involved in synaptic transmission and related to the molecular etiology of Parkinson’s disease (PD): **SCNA (BF7-Chr4)** and **SNCAIP (BF8-ChrZ)** (Table 1).

## Discussion

### Decreased Stress and Reactive Aggression

Our study found intensified selection in the WBM relative to BF on regions comprising genes linked to the hormonal regulation of stress and aggressive responses. The gene **TBX19** (**WBM1-Chr1**) encodes a transcription factor that marks pituitary cell lines that will later express pro-opiomelanocortin (POMC), the peptide that elicits adrenocorticotropic hormone (ACTH) release [46]. Changes in **TBX19** are suggested to underlie tameness and timidity in Chinese indigenous pigs [47]. Though **MARCHF11** (**WBM3-Chr2**) function in the pituitary remains unexplored, emerging findings suggest that its brain expression is regulated by social and stress-related cues tied to reproduction [48]. Key to this process is vasopressin, a hormone synthesized within the hypothalamus and stored in the posterior pituitary. Vasopressin is released to target brain regions, where it has an indirect inhibitory effect on gonadotropin-releasing hormone through the potentiation of ACTH and stimulation of cortisol secretion [49]. **MARCHF11** expression is sexually dimorphic in midbrain vasopressin-responsive neurons, which are activated by prosocial stimuli (e.g., the presence of a female mouse, leading to mating or huddling) but not by antagonistic social stimuli (e.g., the sight of a male mouse, leading to fighting or drawing back) [48]. In songbirds, neurons expressing the avian homolog of the mammalian vasopressin, vasotocin, exert effects on sex-specific behaviors, pair bonding, gregariousness, and aggression [50, 51]. These observations implicate **MARCHF11** in sex-specific reproductive physiology and behavior that discriminates between stress conditions, as regulated by vasopressin/vasotocin. **AKR1D1** (**WBM2-Chr1A**) encodes a steroid A-ring reductase, 5β-reductase, which participates in bile acid synthesis and regulates hormonal action in the liver, including glucocorticoid-mediated effects on carbohydrate and lipid metabolism associated with stress responses [52]. The enzyme 5β-reductase is highly prevalent in the brain and pituitary of birds, where its primary function is to inactivate testosterone [53]. In oscine species, steroid metabolism in the brain is linked to structural changes, particularly in the neural circuits that govern song production and modifications in sexual and aggressive behaviors [54]. Altogether, our findings indicate heightened selection pressure on genes associated with the hypothalamic-pituitary-adrenal (HPA) axis function in WBMs relative to their domesticated descendant BFs. This phenomenon likely contributes to the observed differences in aggressive behavior and stress responses between these two groups, aligning with prior studies in mammalian domesticates [15].

### Reduced neophobia in feeding contexts

Controlled observations reveal that BF exhibit decreased levels of neophobia in feeding contexts relative to their wild counterparts, as demonstrated by their lower latency in approaching the food cup in the presence of a foreign object [8]. This result can also be explained by differences in food motivation between BF and WBM, to which our discovery of evolutionary changes in regions encompassing genes implicated in reward aspects of food intake may relate. Changes in **SNCA (BF7-Chr4)** mRNA and protein levels are associated with reward aspects of food intake and social interactions in mammals [55, 56]. *In vivo* studies suggest that the **SNCA** gene product, alpha-synuclein, regulates dopamine (DA) availability in response to the direct action of metabolic hormones in DA centers or downstream from homeostatic signaling in the hypothalamus [56]. Similarly, **SNCAIP (BF8-ChrZ)** gene product, synphilin-1, is implicated in food intake and fat deposition in mammals [57-59] and in hyperphagia, first characterized in transgenic mice overexpressing this gene predominantly in neurons, which unexpectedly manifested obesity resulting from increased food intake, and in the absence of PD-like symptoms [60]. Signatures of selection at **SNCAIP** have been detected in broiler chicken lines divergently bred for abdominal fat content, which supports synphilin-1’s relation to food intake and adiposity as being conserved in avian species [61].

### Increased flexibility in birdsong learning and practice

Birdsong is regulated by dopaminergic innervation originating in the brain’s motivational centers and projecting to specialized telencephalic nuclei, including the striatal region known as Area X and cortical vocal control nuclei, such as HVC (used as a proper name) and the robust nucleus of the arcopallium (RA) [62]. Our study found intensified selection in the BF relative to WBM on regions comprising genes tightly linked to dopaminergic transmission, thus capable of affecting brain circuits highly relevant for song learning and production. The regulation of DA levels within song nuclei relates to corresponding changes in the expression of enzymes involved in DA biosynthesis, like **DDC (BF5-Chr2),** coding for dopa decarboxylase [62]. In male songbirds, **DDC** mRNA levels in the ventral tegmental area (VTA), the origin of the dopaminergic pathways, are negatively correlated with the amount of contact received from a female partner [103]. The gene **SNCA (BF7-Chr4)** negatively regulates DA availability within song control nuclei, where it is constitutively downregulated at both mRNA and protein levels [63, 64]. It has been proposed that overall low levels of alpha-synuclein in these regions may serve as a protective mechanism against protein aggregation and consequent motor deficits during aging [65]. **SNCA** expression is developmentally downregulated during the critical period for song learning within RA and Area X’s adjacent efferent nucleus, the lateral magnocellular nucleus of the anterior nidopallium (LMAN) [64, 66]. In the adult songbird brain, alpha-synuclein is differentially regulated within Area X between vocal practice and performance, with increased levels scaling to the time spent singing alone for practice but not during singing directed to females [67]. Though less is known about synphilin-1’s function within the song circuit, **SNCAIP (BF8-ChrZ)** mRNA expression is significantly downregulated within Area X during song practice [68]. This result is consistent with synphilin-1’s role in regulating the ubiquitin-mediated degradation of alpha-synuclein [69] and suggests this interaction contributes to the modulation of song variability during practice.

We also found signals of selection present only in the BF population in genes regulating axon guidance and connectivity that contribute to the differentiation and specialization of the song control circuit, **COL4A1** and **COL4A2 (BF2-Chr1),** and **SLIT2 (BF6-Chr4). COL4A2** and **COL4A1** gene products show differential expression between song nuclei in male songbird brains and corresponding vestigial reminiscences in female brains in species where song is sexually dimorphic [70]. **COL4A1** expression is particularly enriched in HVC, where it peaks during sensorimotor learning (i.e., 45 days post-hatch in zebra finches) [71]. Although less is known about the specific role of **SLIT2** within the song circuitry, the down-regulation of its paralog SLIT1 in song nuclei RA and HVC and in the brainstem motor nucleus nXIIts is suggested to facilitate the targeting of ROBO-expressing terminals and guide the development of neuronal projections from HVC to RA, as well as direct projections from RA to nXIIts, a connection critical for learned vocalizations [72].

## Conclusion

Results from various avenues of inquiry indicate that a typical recipe to domestication includes the attenuation of selective forces for augmented reactive aggression and the intensification of ones leading to dynamic modulation of motivation and reward sensitivity. Our findings suggest that the same principles apply to tame a songbird. Major selective sweeps in the BF comprise genes essential for DA synthesis, availability, and response in the songbird brain, thus highly relevant in the specialization and function of brain nuclei dedicated to song. Our scans also reveal selection signals specific to the BF in genes causally linked to pigmentation differences in other animals, which could similarly explain BF color morphs.

## Figures and Tables

**Table 1.** Candidate genes for the domestication syndrome within ROIs

ROI	Sweep Signal		Genes	Gene product; function*
	BF	WBM		
**BF1-Chr1**			HERC2, OCA2, GABRG3	P protein; melanogenesis [41]
**BF2-Chr1**			COL4A2, COL4A1	Collagen alpha-2 and alpha-1 (IV) chains; cell adhesion and signaling [73]
**BF3-Chr1A**			WNT7B	Protein Wnt-7b; neural crest specification and neural cell dendritic arborization [74]
**BF4-Chr2**			CDH9	Cadherin-9; synapse assembly [75]
**BF5-Chr2**			DDC	Dopa decarboxylase; dopamine biosynthesis [76]
**BF6-Chr4**			SLIT2	Slit homolog 2 protein: neural cell axon guidance [77]
**BF7-Chr4**			SNCA	Alpha-synuclein; synaptic transmission [78]
**BF8-ChrZ**			SNCAIP	Alpha-synuclein interacting protein-1; synaptic transmission [79]
**WBM1-Chr1**			TBX19	T-box transcription factor TBX19; pituitary gland development [46]
**WBM2-Chr1A**			AKR1D1	Aldo-keto reductase family 1 member D1; steroid metabolism [52]
**WBM3-Chr2**			MARCHF11	E3 ubiquitin-protein ligase MARCHF11; cell differentiation [80]
**WBM4-Chr3**			SMOC2	SPARC-related modular calcium-binding protein 2; craniofacial morphology [34]

* Noted function of relevance for this study

## Data Availability

The raw sequencing data generated during the current study is available in the SRA NCBI repository under accession code [TBD]. Other datasets generated and/or analyzed are available in the GitHub repository, https://github.com/madzafv/SongbirdDomesticationGenomics. The corresponding author can provide any additional information required to reproduce the results reported in this study upon reasonable request.
